# Complete pathologic response to neoadjuvant icotinib in stage IIIA EGFR-mutant lung adenosquamous carcinoma: A case report

**DOI:** 10.1097/MD.0000000000036214

**Published:** 2024-01-26

**Authors:** Zhongfu Cai, Jishui Huang, Wenliang Dai, Xiaobin Li, Wencong Hong, Youzhi Hong

**Affiliations:** a Department of Respiratory and Critical Care Medicine, The Hospital of Nan'an, Quanzhou, China.

**Keywords:** EGFR-TKI, lung adenosquamous carcinoma, neoadjuvant therapy

## Abstract

**Rationale:**

Radical surgery offers the best chance of cure, it is critical to expand surgery opportunities for patients with early-stage lung cancer to prolong overall survival. However, evidence is still limited regarding the application of neoadjuvant therapy with EGFR-tyrosine kinase.

**Patient:**

The patient reported here was a 53-year-old woman with right lower lung adenosquamous carcinoma.

**Diagnoses:**

The lung cancer was staged as T3N1M0. Tumor genotype disclosed EGFR Exon19 c.2235-2249de p.E746-A750del.

**Intervention:**

After neoadjuvant treatment with icotinib, she underwent thoracotomy and achieved pathological complete response.

**Outcomes:**

She is currently receiving adjuvant icotinib therapy without recurrence or metastasis during 18-month follow-up.

**Lessons:**

Our case indicated that the feasibility of neoadjuvant icotinib in EGFR-mutant lung adenosquamous carcinoma.

## 1. Introduction

Lung cancer is one of the most common malignancy in China, ranking first in morbidity and mortality. Although some improvements have been seen in the prognosis of lung cancer patients by the development of biomedical research and next-generation sequencing technology, the 5-year overall survival rate is approximately 15%, indicating unmet need for patients diagnosed with non-small cell lung cancer (NSCLC). Radical surgery offers the best chance of cure, therefore, it is critical to expand surgery opportunities for patients with early-stage lung cancer to prolong overall survival. The last 2 decades have witnessed fast evolving treatment landscape of metatastic NSCLC with a biomarker-driven approach, more recently, targeted therapies have been extended to the early-stage setting with approval of several EGFR-tyrosine kinases (TKIs) as adjuvant therapy. However, evidence remains insufficient regarding the application of neoadjuvant EGFR-TKIs in lung cancer, especially for adenoaquamous carcinoma. Herein, we reported a case who achieved pathological complete response after neoadjuvant icotinib for stage IIIA adenoaquamous carcinoma.

## 2. Case report

**Figure 1.**
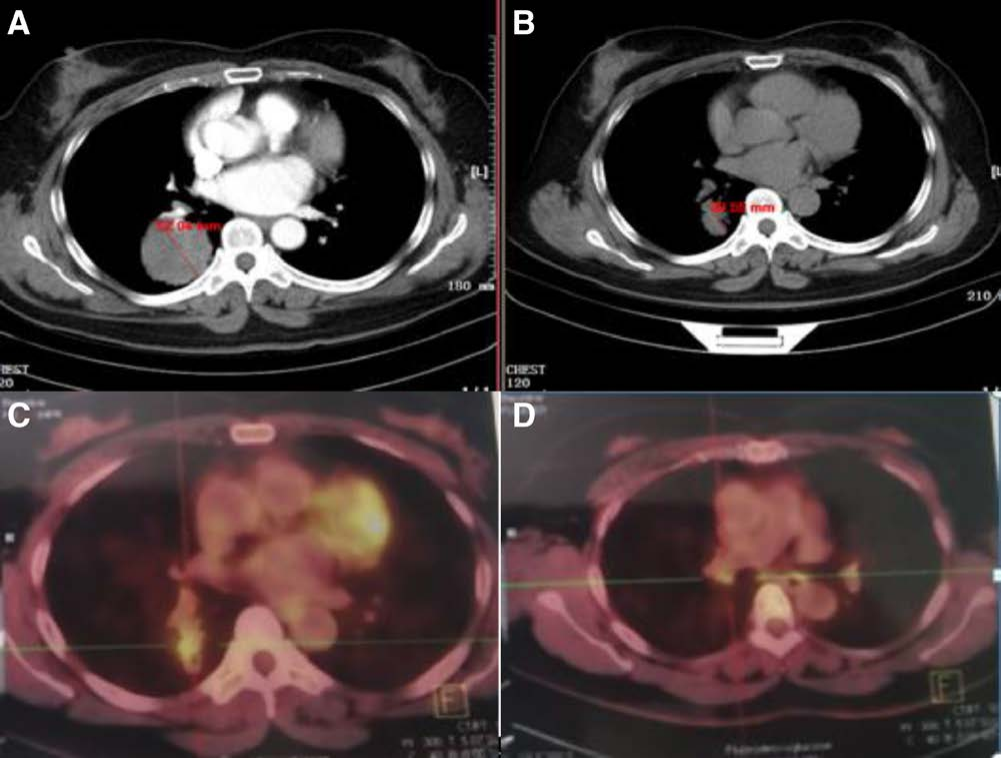
Radiological images (computed tomography and 18F-fluorodeoxyglucose/positron emission tomography), showing the changes of lung masses during treatment course. Pretreatment (A), one-month treatment (B) and 3-month treatment (C and D) with neoadjuvant icotinib.

A 53-year-old woman consulted for persistent cough and expectoration on September 1st, 2021, without history of smoking and alcohol use nor family history of oncology. A contrast-enhanced computed tomography (CT) scan revealed a mass of 5.6 × 5.3 cm with lobulated edge located in the right lower lobe, as well as enlarged hilar lymph nodes (Fig. 1). No additional site of active disease was seen by brain magnetic resonance imaging, abdominal CT, ultrasound, or emission computed tomography (ECT) of the bone. Serum tumor markers were evaluated included NSE, CA-125, CA19-9, and carcinoembryonic antigen, the level of which was 24.32 ng/mL, 85.8 ng/mL, 578.1 ng/mL, and 167.64 ng/mL, respectively. The peripheral total white blood cell count, blood chemistry, coagulation profile, lung function, and echocardiography were all normal. Lung biopsy via bronchoscopy was performed on September 3rd, 2021, and the pathological examination revealed adenosquamous carcinoma. The immunohistochemistry showed the following staining patterns: Napsin-A (+), thyroid transcription factor-1 (+), cytokeratin 7 (+), P40 (+), cytokeratin 5/6, and Ki67 (30%+). Tumor genotype (Repugene Technology, Hangzhou, China) disclosed EGFR Exon19 c.2235-2249de p.E746-A750del with a mutant abundance of 21.7% while lack of expression of programmed death ligand 1. Based on the pathological examination and imaging tests, the patient was diagnosed with lung adenosquamous carcinoma (right, T3N1M0, stage III). The patient was evaluated by a multidisciplinary team who decided to administer neoadjuvant chemoradiotherapy followed by surgery. However, the patient and her family refused this treatment, icotinib was then given 125 mg thrice per day on September 18th, 2021. Partial response was achieved after 4-week treatment of icotinib following the RECIST version 1.1 criteria with remarkable shrinkage of the right lower lobe lesion (CT: 3.2 × 2.9cm, October 22th, 2021, Fig. 1A), and the tumor continued to shrink (positron emission computed tomography: 2.9 × 1.7 cm, SUVmax: 4.5, December 15th, 2021, Fig. 1B). Additionally, positron emission computed tomography showed enlarged and partly calcified mediastinal lymph nodes at 10R (0.7 × 0.5 cm) and 4R (1.4 × 0.7 cm) level (Fig. 1C and D). Declining levels of serum tumor markers were also seen, the value of CA-125, CA19-9, and carcinoembryonic antigen was 8.2 ng/mL, 42.21 ng/mL, and 2.6 ng/mL, respectively. A R0 right upper lobectomy and mediastinal lymph node dissection was performed on January 7th, 2022. The specimen showed a pathological complete response (pCR, pT0pN0). The patient was recommended additional 2 years of postoperative icotinib and serum tumor markers were negative after 1-month treatment. The patient is currently receiving therapy without recurrence or metastasis during 18-month follow-up after surgery.

We obtained informed consent for publication of the case.

## 3. Discussion

Neoadjuvant therapy provides opportunities of reducing the tumor bulk, downstaging, suppressing micrometastasis, and assessing on-treatment response to improve the outcome for potentially operable patients. Currently, platinum-based neoadjuvant therapy has been recommended as the stand of care for NSCLC patients by both National Comprehensive Cancer Network and Chinese Society of Clinical Oncology guidelines, while neoadjuvant chemoradiotherapy are also reported in some studies. However, neoadjuvant chemotherapy confers a modest benefit with increased toxicities, there remains urgent need for safe and effective treatment option. Endothelial growth factor receptor (EGFR) mutations are found in up to 40% to 50% of the Asian population, and the treatment landscape of NSCLC was dramatically changed by EGFR-TKIs as systemic and adjuvant treatment. These developments have inspired great interest in using EGFR-TKIs in the neoadjuvant setting. However, evidence is still limited regarding the efficacy, safety, and strategy for neoadjuvant therapy.

Several EGFR-TKIs are commercially available in clinical practice, including first-generation gefitinib, erlotinib, and icotinib, second-generation dacomitinib and afatinib, and third-generation osimertinib and almonertinib, which has been investigated in early-stage NSCLC patients harboring EGFR mutation. The EMERGING-CTONG 1103 trial is the first published randomized clinical trial comparing the efficacy of erlotinib with gemcitabine plus cisplatin as neoadjuvant treatment in stage IIIAN2, EGFR-mutant NSCLC, indicating improved progression-free survival (PFS) and objective response rate (ORR) as well as less toxicities with erlotinib.^[1]^ In another phase 2 study, neoadjuvant erlotinib was associated with higher objective response rate (67.4% [4 complete remission and 25 partial response] vs 44.2% [4 complete remission and 25 partial response]), histological response rate (65.1% [8 grade I and 20 grade II] versus 41.9% [3 grade I and 15 grade II]) and resection rate (90.7% versus 83.7%).^[2]^ Wang et al reported an retrospective analysis of 67 stage IA-IIIA NSCLC patients treated with neoadjuvant icotinib, which revealed an ORR of 42.1% in 38 patients harboring an EGFR mutation, indicating icotinib could induce clinical response with minimal toxicity.^[3]^ Collectively, first-generation EGFR-TKIs displayed favorable efficacy in terms of downstaging and improved PFS as well as decreased toxicity.

Osimertinib is the first approved third-generation EGFR-TKI that could overcome resistance induced by first- or second-generation EGFR-TKIs and has shown a survival benefit in NSCLC patients with early-stage and metastatic disease. The role of third-generation EGFR-TKIs continue to be investigated for nonmetastatic disease in preoperative contexts. In the NEOS trial, 40 patients with stage II-IIIB, EGFR-mutant NSCLC were enrolled and treated with neoadjuvant osimertinib, out of 38 assessable patients, the ORR and DCR was 71% and 100%, respectively, with 5% achieving pCR.^[4]^ Seven (41.2 %) of the 17 resected patients with N2 disease at baseline experienced downstaging to N1 or N0 after neoadjuvant osimertinib. Additionally, it was notable that the R0 resection rate was 95%, especially in a population that approximately 60% patients had stage IIIA or IIIB disease.^[4]^ Other series involving neoadjuvant osimertinib showed objective response rates ranging from 46% to 100% and variable major pathological response (MPR) rates from 15% to 75%.^[5–7]^

Although there are data to reveal favorable response of neoadjuvant EGFR-TKIs, however, most studies failed to show overall survival benefits. EMERGING CTONG1103 has reported no difference of overall survival between the erlotinib and the chemotherapy arm (45.8 vs 39.2 months) in EGFR-mutant patients with stage IIIAN2 disease.^[1]^ Similarly, in another phase II study with neoadjuvant gefitinib, despite significantly prolonged disease-free survival in patients with MPR than those without, the overall survival did not differ between the 2 populations.^[8]^ Whether neoadjuvant treatment with the third-generation EGFR-TKI would improve overall survival remains an open question, which could be further explored in ongoing and future studies.

Studies also have explored the combination of EGFR-TKI with chemoradiotherapy in neoadjuvant setting. Two-month afatinib followed by neoadjuvant chemoradiotherapy showed a MPR of 57.1% with one pCR (14.3%). Seven patients underwent surgery and the remaining patients received afatinib, displaying a median PFS of 34.6 months and a 2-year overall survival of 85%.^[9]^ These findings provide new insight for EGFR-TKIs as neoadjuvant treatment but require further clinical validation.

In the present case, on the basis of literature review, considering the availability and cost-effectiveness, as well as the patient’s unwillingness to accept chemoradiotherapy, pre-operative icotinib was administered, showing a rapid response within one month, along with R0 resection, pathological complete remission, normal level of tumor markers, and an event-free survival of 18 months.

So far, neoadjuvant treatment with EGFR-TKIs has not been recommended as standard of care in the absence of confirmatory studies. The improved clinical outcomes found in the literature and the positive result obtained in the present case suggest the utility of neoadjuvant EGFR-TKI in NSCLC, even in lung adenoaquamous carcinoma patients harboring EGFR mutation. However, several questions remain to be explored, including determining, whether MPR can be transferred into survival benefit, whether outcome can be improved by more duration or exposure of treatment, the feasibility of combining EGFR-TKI and chemotherapy, radiotherapy, or immunotherapy in neoadjuvant setting, the optimal timing of surgery after neoadjuvant therapy, and the subsequent treatment after surgery.

## Author contributions

**Conceptualization:** Zhongfu Cai, Youzhi Hong.

**Data curation:** Jishui Huang, Wencong Hong.

**Visualization:** Wenliang Dai, Youzhi Hong.

**Writing – original draft:** Zhongfu Cai, Wenliang Dai, Youzhi Hong.

**Writing – review & editing:** Jishui Huang, Xiaobin Li, Wencong Hong, Youzhi Hong.
